# Correlations of Imaging and Therapy in Breast Cancer Based on Molecular Patterns: An Important Issue in the Diagnosis of Breast Cancer

**DOI:** 10.3390/ijms25158506

**Published:** 2024-08-04

**Authors:** Oana Maria Burciu, Ioan Sas, Tudor-Alexandru Popoiu, Adrian-Grigore Merce, Lavinia Moleriu, Ionut Marcel Cobec

**Affiliations:** 1 Doctoral School, Faculty of Medicine, “Victor Babes” University of Medicine and Pharmacy Timisoara, 300041 Timisoara, Romania; 2 Department of Functional Sciences, Medical Informatics and Biostatistics Discipline, “Victor Babes” University of Medicine and Pharmacy, 300041 Timisoara, Romania; 3 Department of Obstetrics and Gynecology, “Victor Babes” University of Medicine and Pharmacy, 300041 Timisoara, Romania; 4 Department of Cardiology, Institute of Cardiovascular Diseases, 300310 Timisoara, Romania; 5 Clinic of Obstetrics and Gynecology, Klinikum Freudenstadt, 72250 Freudenstadt, Germany

**Keywords:** breast cancer, molecular subtypes, histologic classification, mammography, ultrasonography, MRI, guided biopsy

## Abstract

Breast cancer is a global health issue affecting countries worldwide, imposing a significant economic burden due to expensive treatments and medical procedures, given the increasing incidence. In this review, our focus is on exploring the distinct imaging features of known molecular subtypes of breast cancer, underlining correlations observed in clinical practice and reported in recent studies. The imaging investigations used for assessment include screening modalities such as mammography and ultrasonography, as well as more complex investigations like MRI, which offers high sensitivity for loco-regional evaluation, and PET, which determines tumor metabolic activity using radioactive tracers. The purpose of this review is to provide a better understanding as well as a revision of the imaging differences exhibited by the molecular subtypes and histopathological types of breast cancer.

## 1. Introduction

Breast cancer (BC) holds a significant role in the 21st century epidemiological, ethnic, and environmental global picture. Carcinogenesis is a complex process that can affect every cell, tissue, or organ in the body, manifesting in a variety of cancer types. The major physiopathological mechanisms appear to be evasion of apoptosis, high division capacity, pronounced angiogenesis, resistance to anti-growth factors, induction of growth signals, and metastasizing capacity [1].

Cardiovascular diseases (CVD) and cancer are the leading causes of premature death in 127 countries (CVD leading in 70 countries and cancer leading in 57). However, based on recent surveys, the latter may surpass the former in most countries during the course of this century [2].

In this international context, BC has established itself as the leading cause of cancer worldwide, surpassing lung cancer and other types, representing 11.7% of all cancer cases. Data gathered by the World Health Organization (WHO) and recent studies from 2021 and 2022 indicate over 2,2 million BC diagnoses in women and over half a million deaths globally, meaning that around 15–16% of cancer deaths and 25–30% of cancer cases are attributed to BC [3,4,5]. If this trend continues, specialists fear that 20 years from now, due to various reasons such as processed foods and population growth, there will be over 3 million new BC cases every year [5,6].

The overall 5-year survival rate for BC presents great variations that range from 80% in more developed countries to 50% in less developed countries. These variations further strengthen the association between BC and the extent of socio-economic development [7]. Previous studies showed that 5-year BC survival rates surpassed 90% in some regions of the United States, in contrast to the survival rates in South Africa and India, which were estimated at around 40–50% in South Africa and 66–70% in India [4,8].

Considering the age of incidence for BC as well as the 5-year survival, data collected from practice and multiple studies suggest that age is considered the most important risk factor in developing this disease. Up to 95% of cancer cases occur in women aged 40 or older. The median age at the time of diagnosis has been found to be 61 years old [9,10].

Ethnicity also plays an important part in this pathology. Several early studies stated that African American and Hispanic women have inferior BC survival rates compared to Caucasian women, even though non-Caucasian women have a lower incidence of developing the disease. Asian or Asian/Pacific Islander (API) ethnicity was associated with better survival outcomes in multivariable analyses. African American ethnicity is usually associated with an increased frequency of the aggressive triple-negative BC (TNBC) subtype and is considered to be an independent predictor of a poor outcome, a situation also observed in Australian aboriginal communities, Malay ethnicity, and minority immigrant populations [11].

**Figure 1:**
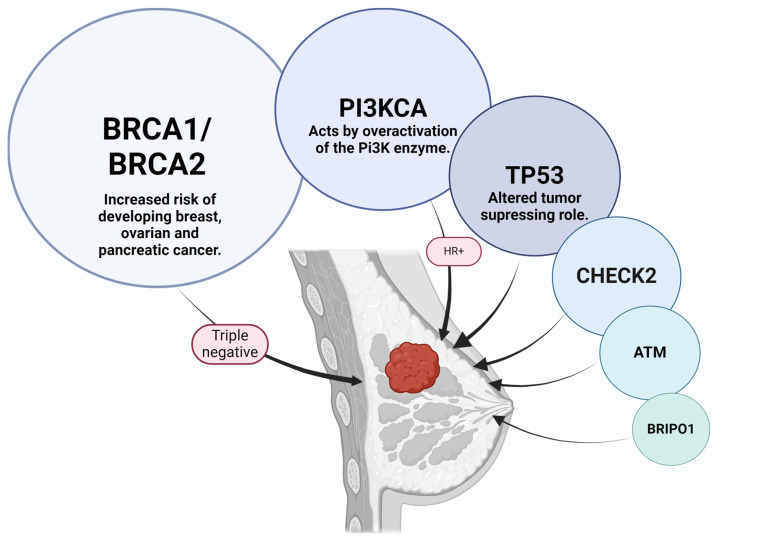
Gene mutations that can increase the risk of breast cancer. Abbreviations: BRCA = breast cancer gene; PI3KCA = phosphatidylinositol-4,5-bisphosphate 3-kinase, catalytic subunit alpha; TP53 = tumor protein p53; CHECK2 = checkpoint kinase 2; ATM = ataxia-telangiectasia mutated; BRIP1 = BRCA1 interacting protein.

Regarding the genetic mutations that are known to increase the risk of developing BC, the European Society of Breast Cancer Specialists (EUSOMA) estimates that 3% of all BC cases are caused by an underlying deleterious mutation of the BRCA1 or BRCA2 genes [12]. BRCA genes play a very important part in the BC genetic etiology because of their role in repairing damaged DNA. Furthermore, mutations of the BRCA genes are also associated with an increased risk of ovarian cancer. In the case of clinically significant mutations, the estimated lifetime risk is about 80% for BC and 40–65% for ovarian cancer. Ovarian cancer is considered another public health problem known to have a poor prognosis, ranked as the seventh most common cancer type in women in terms of incidence and mortality worldwide [13,14,15]. Recent research has pointed out that BRCA1 mutation carriers with BC had more chances of developing TNBC than BRCA2 mutation carriers or non-carriers [16]. A smaller percentage of BCs are caused by TP53 mutations or, in rare cases, by moderate penetrance alleles such as CHEK2, ATM, and BRIP1, as shown in Figure 1 [17,18]. 

Other less frequent mutations or proliferation markers have been correlated with certain molecular BC subtypes. Antigen Ki-67, also known as Ki-67 or marker of proliferation Ki-67 (MKI67), is a protein whose expression reliably correlates with cancer proliferation [19]. Immunohistochemical evaluation of this marker has been used for years to indicate cancer prognosis, outcome, and treatment response.

PIK3CA gene mutations can lead to the overactivity of the PI3K enzyme, promoting the growth of cancer cells. Even though changes in the PI3K gene can cause a variety of cancers, when referring to BC, PI3K mutation or amplification and other aberrations in the PI3K signaling pathway can often be observed in hormone receptor-positive (HR+) BC. In recent years, PIK3CA inhibitors, such as alpelisib, have shown significant progress in HR+/HER2-negative (HER2-) metastatic BC [20].

## 2. Diagnostic Methods

Over the past decades of research, every clinical trial conducted concluded that an early diagnosis and prompt therapeutic action are crucial in improving the survival of patients with BC. An early diagnosis will positively impact the effectiveness of the treatment and the prognosis of the disease. It has been proven in several studies that T1 tumors with a diameter of less than 2 cm have a 10-year survival of approximately 85%, while T3 tumors, essentially the result of delayed diagnosis, have a 10-year survival of less than 60% [21,22].

A lump situated in the breast tissue can be assessed by using manual clinical examination of different imaging techniques, such as mammography (MG), ultrasound (US), magnetic resonance imaging (MRI), scintimammography, single photon emission computed tomography (SPECT) and positron emission tomography (PET), and guided biopsy (usually guided by ultrasound). Out of all of these methods, MG and US are the most convenient for screening, while MRI has proven to increase overall specificity and sensitivity in some cases. Each method has its own advantages and disadvantages, which will be discussed next [23].

### 2.1. Clinical Breast Examination (CBE)

**Figure 2:**
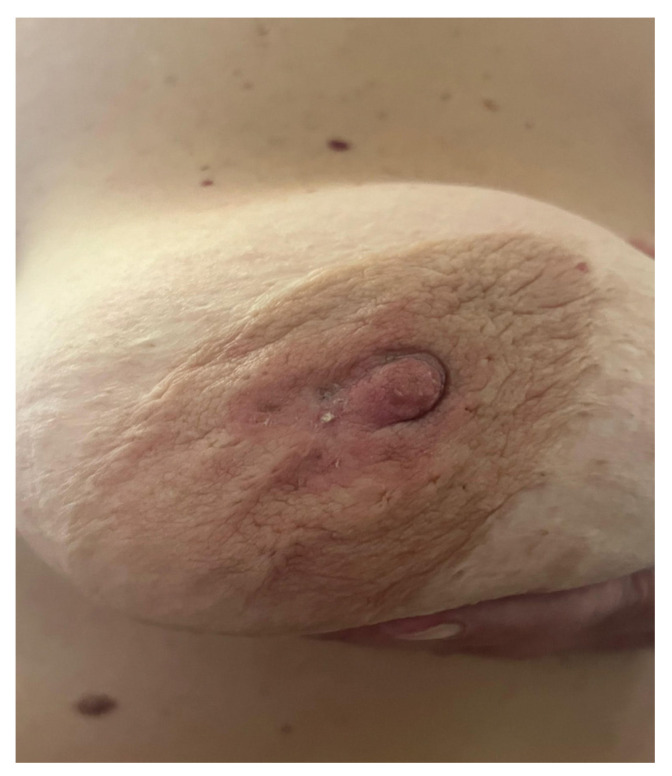
Clinical examination of the breast reveals nipple retraction and epidermal infiltration.

A recent retrospective cohort study has shown that the sensitivity of CBE was found to be 88.9%, compared to 89.8% for MG and 95.1% for US [24]. CBE continues to demonstrate its utility, especially in regions where BC awareness and screening are lacking and healthcare resources are limited; the use of this method is portrayed in Figure 2 in a patient with breast cancer displaying nipple retraction and epidermal infiltration [25].

### 2.2. Mammography (MG)

MG, or Full Field Digital Mammography (FFDM), is widely used for BC diagnosis and screening and is considered the gold-standard screening method [26].

**Figure 3:**
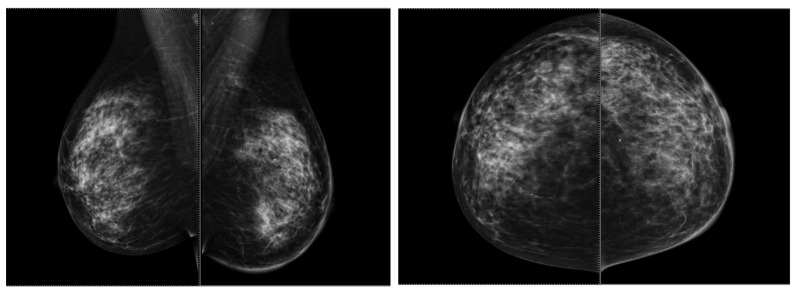
Multiple architectural distortions throughout the whole left mammary gland with enlarged homolateral lymph nodes, spiculated lesions in the superior external quadrant, BIRADS SCORE of 6.

Contrast-enhanced mammography (CEM) and digital breast tomosynthesis (DBT) are the most commonly used diagnostic tools nowadays and represent adjuncts to FFDM; a case that accentuates their utility is presented in Figure 3. No significant differences regarding cancer detection were found between them [22,27].

MG shows a sensitivity of 78% and specificity of 99%, diminishing to 70% and 91%, respectively, as breast density increases [27,28]. In these cases, DBT and CEM have proven to be superior. Other studies demonstrated that up to 80% of cancers misdiagnosed using MG were located in dense breasts [29].

Current data suggest that annual MG screening could lower mortality by 30–40% [22].

### 2.3. Ultrasonography (US)

Even though mammography is considered the gold standard for screening, ultrasound is a very helpful tool due to its many well-known advantages [30]. US sensitivity is not affected by a higher density of the breast, in contrast to mammography. A woman with negative mammography and dense breast tissue who undergoes a screening ultrasound can achieve better detection rates [29].

High sensitivity was observed in small breast lesions (2 cm or smaller) when using US combined with MG. Thus, combining both imaging tools resulted in higher sensitivity compared to using either modality alone [29].

Several studies and reviews reported slightly higher recall rates and biopsy rates for US relative to MG; however, the differences were not significant. Furthermore, US screening has been shown to be comparable to, and in some cases even superior to, MG in women with high breast density in terms of sensitivity, specificity, and cancer detection rate [31].

Multiple studies showed that luminal A tumors are usually smaller in size, while basal-like type tumors are mostly larger and more likely to have a regular shape. Luminal A and luminal B types are typically not circumscribed, whereas basal-like types are described as circumscribed masses. Regarding the lymph node criteria, luminal A type tends to have normal-appearing lymph nodes, but luminal B type, HER2-positive (HER2+) type, and basal-like type tend to have abnormal ones [32].

In recent years, contrast-enhanced ultrasound (CEUS) has played an important role in the differential diagnosis of the molecular types of BC because of their different contrast enhancement patterns and different perfusion parameters. The contrast enhancement pattern of a triple-negative tumor may be easily overlooked because it typically shows a well-defined border, resembling the contrast enhancement pattern of benign tumors. The HER2-enriched subtype usually presents centripetal enhancement, whilst the luminal epithelium subtype is often characterized by a low perfusion enhancement pattern [33].

Mammography, ultrasound, and Doppler can be and should be used together in order to maximize diagnostic accuracy. Recent imaging reports indicate that the luminal A subtype usually appears as a non-circumscribed mass with spiculated margins, posterior shadowing, and very poor vascularity. The luminal B subtype is also non-circumscribed and has posterior shadowing but is rich in vascularity. The HER2-enriched subtype can be recognized by its microlobulated margins, microcalcifications, and posterior mixed acoustic pattern. Finally, the triple-negative subtype is well-circumscribed with posterior enhancement and no vascularity [34].

### 2.4. Magnetic Resonance Imaging (MRI)

Numerous studies have stated the superiority of MRI compared to other imaging methods, with a sensitivity of 95% in detecting breast malignancies. MRI is currently the most precise imaging modality to assess the response to neoadjuvant chemotherapy. It is also highly accurate in evaluating especially triple-negative and HER2+ tumors. The use of preoperative breast MRI remains controversial. Although it plays an important role in delineating the extent of the primary tumor and diagnosing multicentric and contralateral disease, studies have shown that the use of preoperative MRI also leads to an increased number of mastectomies [35].

In a recent observational retrospective study, the addition of MRI to US and MG resulted in a significantly improved sensitivity, from 93.3% to 98.2%. The same study reported that the use of MRI did not significantly impact the mastectomy rate, contrary to the findings of several prior studies [29].

### 2.5. Guided Biopsy

BIRADS 4 (suspicious) and BIRADS 5 (very suspicious) lesions are considered primary indications for breast biopsy. A situation in which guided biopsy is not imperative but should be taken into consideration is when a BIRADS 3 is persistent on a US examination even after 6 months. The patient will usually request a guided biopsy in order to receive an anatomopathological verdict [36].

Guided breast biopsy can be performed using US, mammogram (stereotactic), or MRI, depending on the visibility of the lesion, the preference of the doctor, and other factors. US is, however, the most accessible and commonly used modality [37].

In a study of 768 patients, for the evaluation of several diagnostic tests—MG, MG + US, or US + elastography (ES)—histopathological examination, which provides a definitive diagnosis, was used. When compared with histopathology results, MG + US sensitivity (99.26%) was higher than that of MG (97.73%) and that of US + ES (88.46%), while the specificity was 81.73% for MG alone, 44.37% MG + US, and 67.8% for US + ES; these aspects are also presented in Table 1, portraying the sensitivity and specificity of each method and combinations between them [38].

A cohort study suggested a higher risk of mortality associated with core needle biopsy (CNB) compared to fine needle aspiration (FNA) in women without radiotherapy, probably due to a higher risk of tumor seeding [39]. The mortality risk between FNA and CNB was similar in women undergoing radiotherapy (radiotherapy is capable of destroying locally malignant cells) [39]. A higher rate of distant metastasis in CNB patients compared to FNA patients was reported in a previous study [40].

In BC patients that underwent radiotherapy, excision biopsy displayed a better survival rate in comparison to percutaneous needle biopsy, results that challenge the global trend encouraging surgeons to move away from excision biopsy in order to avoid unnecessary surgery and reduce costs and morbidity [39].

## 3. Discussion

### 3.1. BC Screening, Including Tumor Biology Manifestations in Laboratory Results 

Having now discussed the methods of detection regarding BC, in this section, we will shift our focus toward the screening of this disease, exploring how often the screening should be done, why one method is superior to another, and why no method is perfect. Even though the American Society of Clinical Oncology does not currently recommend the use of tumor markers for screening, diagnosis, or detecting BC recurrence, several biomarkers, such as CA-125, CEA and CA15-3, serve in the management of patients with BC and because of their role as predictive indicators, they help with disease monitoring. Levels of several other markers, such as AFP, FT4, TSH, and PTH, were studied in comparison to CA 15-3 levels in patients diagnosed with BC within a specific cohort; however, no statistically significant associations were identified [41].

As previously mentioned, mammography is currently the gold standard for BC screening due to its accessibility, minimal radiation exposure, and high sensitivity. Mammography screening has proven its efficiency by reducing BC mortality by up to 40% in women aged 40 or older. Therefore, international guidelines advise annual mammography starting at the age of 40 [42].

A 10-year retrospective study compared a cohort of screened women aged 40–49 years who had a 5-year disease-free survival of 94% and overall survival of 97% with unscreened counterparts who had a 5-year disease-free survival of 71% and overall survival of 78% to emphasize the importance of screening [43]. A large cohort study of 549 091 women from Sweden, representing 30% of the screening-eligible population, reported a 41% reduction in the incidence rate of BCs that were fatal within 10 years after diagnosis and a 25% reduction in the rate of advanced BC among screened women [44]

Another useful tool in BC screening is US. Even though US is usually regarded as the second most preferred method in BC screening, there is growing interest in evaluating its performance as a primary screening tool and its effectiveness as a supplemental screening method following a negative MG. 

The results from a meta-analysis of 23 studies published between 2003 and 2018 suggested that supplemental US screening could detect occult cancers missed by MG, while the primary US is comparable in terms of sensitivity, specificity, cancer detection rate, and biopsy rate to MG in women with dense breasts but has higher recall rates [32].

A proposed 10-year follow-up study from Germany aims to investigate the effectiveness of the German mammography screening program in reducing mortality in women aged 50 to 69. The study focuses on one of the major harms mammography screening has, namely overdiagnosing, including subsequent treatment of the overdiagnosed cases [44,45]. More studies have highlighted the issue of overdiagnosis, indicating that the current screening programs in the UK likely achieve only a 20% reduction in mortality for women aged 55–79 while being associated with increased rates of anxiety and discomfort due to false-positive results [45,46].

Another approach emerging in the literature is the risk-stratified approach. In risk-stratified screening, individualized risk assessment determines screening intensity and interval, the age of the first screening, the imaging method, or even the decision not to screen. This concept takes into account family history, breast density, and reproductive factors, as well as demographic, clinical, and genetic parameters. Further research is needed to establish the benefits and potential harms of risk-stratified screening [47].

### 3.2. Correlations between Histological BC Types and Subtypes and Their Genomic Profile

There are over 20 histopathological types of BC. Histopathologists evaluate the limitation of the tumor to the epithelial component or stromal invasion, its origin in mammary ducts or lobes, cell type characteristics, cell count, immunohistochemical profile, and architectural features [48,49].

**Figure 4:**
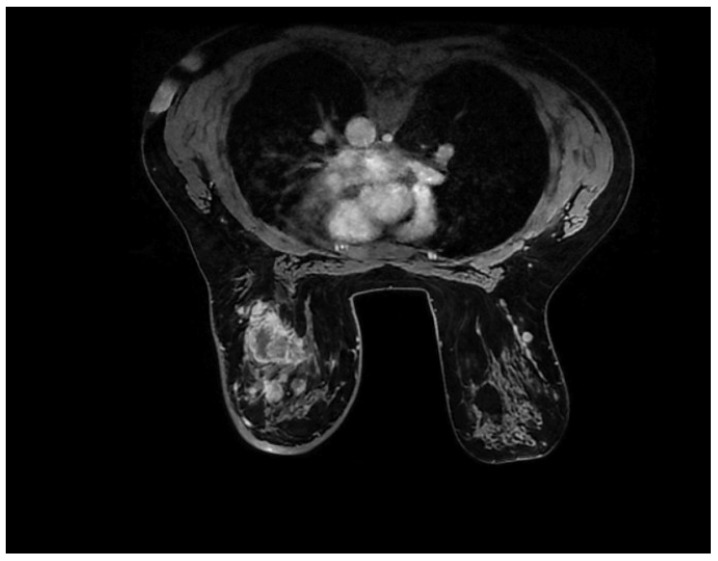
Breast MRI with contrast. In the left breast, a large spiculated lesion with central necrosis and rim enhancement pattern is visible, along with multiple smaller lesions and contralateral extension in the right breast.

Approximately 80% of BCs are invasive ductal carcinomas (IDC). The second most common histological type of invasive BC is invasive lobular carcinoma (ILC), accounting for 5–15%. ILC is different from IDC in that it tends to be more multifocal or multicentric, larger in size, and with a lower histological grade pattern displayed in a patient with ILC histologic type in Figure 4 [50].

IDCs are divided into two subgroups, namely, no specific type (NST), which lacks sufficient morphological characteristics to be classified as a specific histological type, and specific type, which presents sufficient characteristics [49]. According to the WHO classification, the most common specific subtypes are invasive lobular, tubular, cribriform, metaplastic, apocrine, mucinous, papillary, micropapillary carcinoma, carcinoma with medullary, neuroendocrine, and salivary gland/skin adnexal type features [51,52].

In 3–5% of cases, both lobular and ductal morphology can be seen, with these cases of BC regarded as mixed ductal-lobular carcinoma (MDLC) [52,53].

BCs have long been classified based on their histology and genomics. A very interesting study conducted by Thennavan A. et al. aimed to offer a unifying scheme for the molecular, histological, and biological information on BCs. By analyzing an extensive cancer genomic database, the goal was to define the transcriptomic and genomic profiles of the following rare histological types: cribriform, micropapillary, mucinous, papillary, metaplastic, and invasive carcinoma with medullary pattern. They demonstrated the applicability of their research through a predictive model that can detect mucinous histologic types among cancers of other organ systems [54].

### 3.3. Correlations between BC Histological Types and Subtypes and Imaging Features

In this subsection, the aim is to correlate the well-known histological types and subtypes of BC (IDC, ILC, and MDLC) and the different histological grades (1, 2, and 3) with aspects observed on MRI images based on recent studies and original papers; the key aspects are presented in Table 2. A standard multiparametric MRI (mpMRI) protocol will include unenhanced sequences (T2 weighted and DWI), followed by a series of pre and post-contrast T1-weighted acquisitions. mpMRI, contrast-enhanced sequences, and DWI have proven to increase diagnostic accuracy in BC [55].

In one study on MRI examination, most of the invasive lesions appeared as mass enhancements (80.8%), whereas ductal carcinoma in situ (DCIS) appeared as a non-mass enhancement (86.7%). DCIS appeared as isointense on T2-weighted images, with non-mass enhancement (areas that show enhancement without a mass in the pre-contrast sequence), absence of central necrosis, and no axillary adenopathy. There were no associations reported between ILC and MRI characteristics [55].

Other statistically significant correlations were found between invasive NST carcinoma and intralesional necrosis, axillary adenopathy, and multifocal extension of the disease [56].

It appears that both clinical and radiological assessment of ILC is more challenging compared with IDC due to its insidious growth with little or no desmoplastic reaction. Further research is needed to investigate correlations between MDLC and MRI findings [57].

This study from the Faculty of Medicine of Ankara, Turkey, concluded that the T2 signal intensity ratio (SIR) and dynamic curve can facilitate a radiological presumption regarding a certain histopathological pattern. Morphological features and apparent diffusion coefficient (ADC) values were of no particular aid in differentiating histological types and grades. Lower T2 SIR and type 1 dynamic curves have been observed more often in ILC, while IDC and grade 3 demonstrated higher T2 SIR [58].

Mann emphasized through studies the idea that the imaging diagnostic of ILC is challenging due to its diffuse growth compared to IDC [59,60,61].

In their comparative study involving 811 patients based on dynamic and morphologic descriptors, Dietzel et al. observed intermediate to strong early-phase wash-in dynamics and irregular margins in both ILC and IDC [62].

Galati et al. also noted that 86% of their DCIS cases showed non-mass enhancement in MRI contrast-enhanced sequences. Another observation that was supported by previous studies is T2 isointensity in 78% of patients. There was a strong association between DCIS and the absence of axillary adenopathy. MRI features observed in patients with invasive carcinomas NST were mass enhancement, intralesional necrosis, and abnormal axillary lymph nodes. Consistent with prior research, invasive BC mostly presented as a mass with intermediate to low signal on T2-weighted images, reflecting its high cellularity and low water content. Features such as rim enhancement, central necrosis, peritumoral edema, and metastatic axillary lymph nodes were indicative of grade 3 status [55].

### 3.4. Molecular Classification of BC

Understanding the four molecular subtypes of BC discovered to date is crucial. These include (1) luminal A, (2) luminal B, (3) HER2-enriched/overexpressing, and (4) triple-negative, predominantly basal-like cancers. Their distinct behaviors in terms of treatment response, disease progression, incidence, survival, and imaging features are of primary interest in our review. Some classifications also include a fifth type—normal-like tumors [56,63,64].

Estrogen receptor (ER), progesterone receptor (PR), and HER2 are prognostic markers routinely assessed in BC patients due to their predictive value for hormonal and anti-HER2-targeted therapy. ER and PR have the ability to stimulate the growth of both normal and neoplastic breast epithelium in approximately 75% of BCs. When present, they are positive indicators of a good response to hormonal therapy [56].

The most common among all four mentioned subtypes are luminal tumors (60–70%), which are usually ER-positive (ER+). Luminal A tumors are typically low-grade tumors, present a slow growth, and are characterized by a lower Ki-67 expression and lower relapse incidence. Other observations regarding this subtype are a high response rate to hormone therapy and a higher overall survival rate, attributes that confer them the most favorable prognosis among all BC subtypes. Luminal B cancers, on the other hand, have a more unfavorable prognosis. They are higher-grade tumors, often with axillary involvement at the time of diagnosis, and show high Ki-67 expression. They respond well to chemotherapy [65].

ER expression divides BCs into two clusters, namely ER+ and ER-negative (ER-) BCs. Luminal subtypes are, in most cases, ER+ cancers, while the rest of the molecular subtypes are usually ER- [56].

HER2-overexpressing tumors are likely to be high-grade, run an aggressive course and tend to have a poor prognosis. However, since the arrival of anti-HER2-targeted therapy, the patients with this subtype now experience significantly improved prognosis and better chances for a favorable outcome [66].

Finally, the triple-negative subtype (TNBC) has proven to be more aggressive compared to other molecular subtypes, highly recurrent, and with a tendency to target younger women. Prognosis and the evolutive tendency of each of these tumors are displayed in Table 3 [67].

### 3.5. Correlations between Molecular Subtypes of BC and Their Mammographic Features

**Figure 5:**
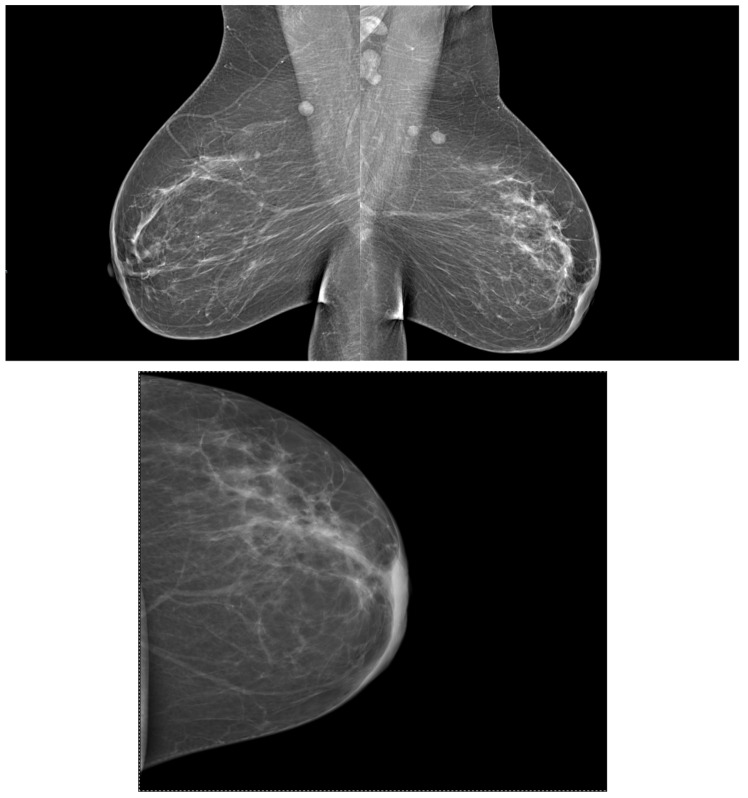
Infiltrative lesion with enlarged homolateral lymph nodes and tegument infiltration. Histopathological exam—invasive ductal breast carcinoma.

**Figure 6:**
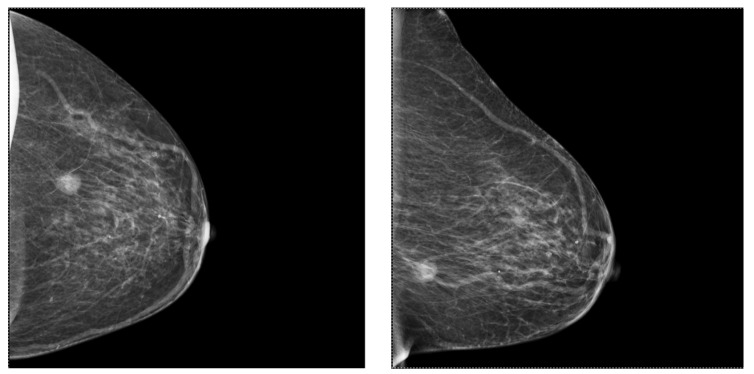
Breast mammogram of the left breast. Discrete hypoechogenic mass just below the areola. Histopathological exam—invasive mucinous carcinoma tip A, SBR Score = I, Nottingham Score = 5.

From a morphological point of view, on a mammogram, all breast tumors present as masses with or without microcalcifications. In Figure 5, a patient with a retroareolar mass is displayed, viewing enlarged homolateral lymph nodes and tegument infiltration, imagistic aspects corresponding to a mucinous breast cancer tip A after biopsy. In a different patient, in Figure 6, we can visualize the malignant lesion via tomosynthesis, that can offer a more detailed and enriched image, than a standard 2D mammogram. In 62.9% of cases, no calcifications are seen. Among all molecular subtypes, HER2-enriched BC subtype and luminal B have been associated with the presence of microcalcifications [68].

In contrast-enhanced mammography (CEM), the average parenchyma enhancement was higher in non-luminal, ER/PR-negative, and Ki-67-positive lesions, as well as in malignant versus benign breast lesions [69].

### 3.6. Correlations between Molecular Subtypes of BC and Their Ultrasonographic Features

**Figure 7:**
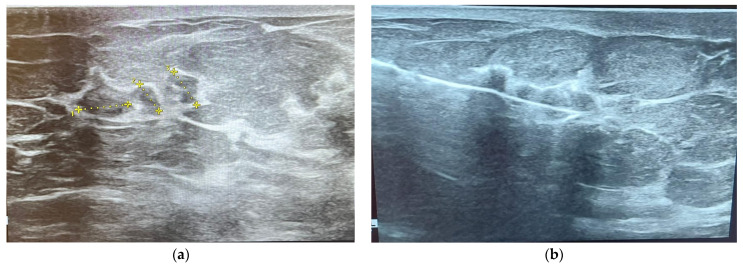
Ultrasound presents a mammary gland with multiple lesions at 2–3 o’clock with spiculated margins and a non-circumscribed shape, with a linear/ductal distribution: (**a**) Measured lesion; (**b**) Insertion of biopsy needle inside the lesions.

On ultrasound assessment, most of the molecular BC subtypes are seen as masses with or without associated calcifications, with irregular or spiculated margins; a patient with such lesions is presented in Figure 7, the first image taken before biopsy and the second picture after the insertion of the biopsy needle. However, some particularities have been noticed. The luminal A subtype has been associated with a peripheral echogenic halo, which can also be observed in triple-negative tumors, while posterior shadowing has been noticed in the HER-2 enriched subtype. Aspects observed in triple-negative tumors include posterior acoustic enhancement or high peritumoral enhancement, architectural distortion, trabecular thickening and lack of microcalcifications in most cases [68,70].

Other interesting observations regarding molecular subtypes of BC and their ultrasonographic features would be the significant correlation between triple-negative BC and circumscribed tumoral margins in contrast with spiculated margins usually observed in hormone-positive tumors. Some of the characteristics of the triple-negative tumors can mimic a benign lesion, the only difference being the tumor size, as larger sizes correlate positively with malignancy. HER2-enriched tumors usually present microcalcifications, increased blood flow signal, isoechoic echo patterns and microlobulated margins. In terms of tumor stiffness on multimodal ultrasonography, luminal A presents high stiffness, whereas luminal B subtype usually presents low stiffness. Another interesting observation regarding ultrasonography is the posterior acoustic shadowing observed in hormone receptor-positive tumors, whereas hormone receptor-negative tumors are usually characterized by posterior acoustic enhancement [70,71].

### 3.7. Correlations between Molecular Subtypes of BC and Their Features on MRI

**Figure 8:**
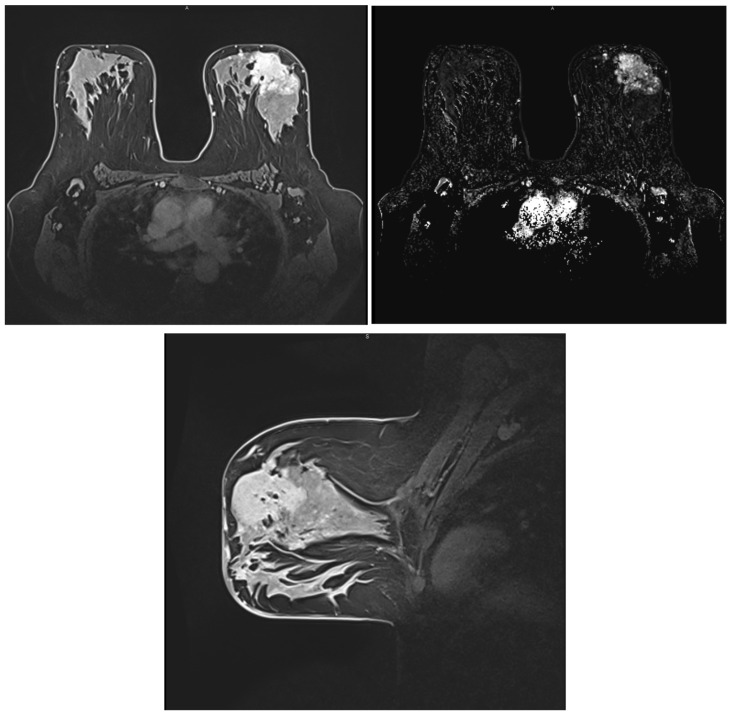
MRI images of BC. Histopathological result—infiltrative mammary carcinoma NST, Nottingham SCORE = 6, associated with areas of comedo-type carcinoma in situ, solid subtype, high grade.

Luminal types are discussed together due to their similar appearance on MRI scans. They usually present as irregularly shaped lesions with spiculated margins, a heterogeneous internal enhancement pattern, and a low to intermediate intratumoral T2 signal, features shown in Figure 8 [72].

HER2-enriched tumors have been associated with an internal heterogeneous enhancement pattern, and similar to luminal types, an intratumoral low T2 signal is usually observed. ADC values are higher compared to the other molecular subtypes, and an early wash-out is commonly seen due to increased vascular endothelial growth factor (VEGF) [73].

Triple-negative tumors have been described as round-lobulated lesions with smooth, circumscribed margins and rim enhancement in the vast majority of cases, a characteristic of high sensitivity in hormone-negative tumors. Triple-negative cancers are usually unifocal lesions with central necrosis. A high T2 signal within the mass is associated with tumoral necrosis, which is a prognostic factor for invasive ductal carcinoma, as well as high ADC/DWI [74,75].

Certain correlations have been reported between tumor margins and the presence of hormone receptors. Oval-shaped lesions have been associated with ER/PR-negative cancers, while irregular, spiculated lesions have been linked with ER/PR-positive cancers. Another interesting observation from various studies concerns the desmoplastic reaction in the adjacent breast tissue, which tends to be lower in high-grade and rapidly growing tumors and more pronounced in low-grade and slowly growing lesions [76].

**Figure 9:**
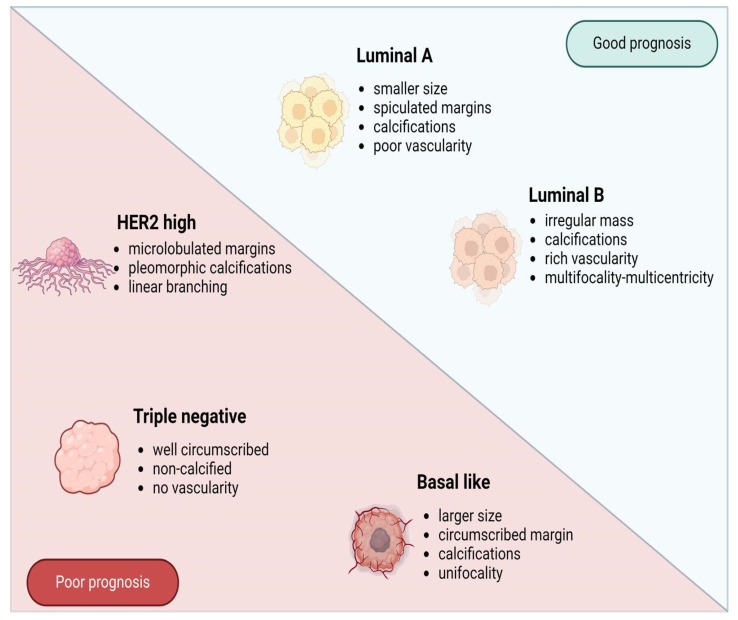
The known molecular subtypes of breast cancer and their corresponding imaging features. Abbreviations: HER2 = human epidermal growth factor 2.

The earliest imaging studies have stated the following observations regarding the four molecular subtypes of BC: non-calcified and circumscribed margins in triple-negative tumors, irregular and spiculated margins in luminal subtypes, and pleomorphic calcifications in the HER-2 enriched subtype, outlined in Figure 9 [77,78]. Since then, multiple studies from various clinics have been conducted, including one reported by Durhan et al. regarding triple-negative BC, which concluded that, in most cases, the tumor is characterized by smooth mass margins, heterogeneous rim enhancement, a persistent enhancement pattern, and very high T2 signal intensity on MRI scans [58].

In one study, two molecular breast techniques—molecular breast imaging (MBI) with Technetium-99m sestamibi and contrast-enhanced mammography (CEM)—were compared with MRI to help with the assessment of locally advanced BC. They concluded that the three imaging techniques showed similar detection of all malignancies. MRI described more nonindex suspicious benign lesions than the other two techniques, resulting in a lower positive predictive value of additional biopsies. All three modalities overestimated the index tumor size, particularly the MRI [79].

PET/CT examinations are another useful tool in determining invasive molecular subtypes of BC by measuring the uptake of fluorodeoxyglucose-18 (FDG), a radioactive sugar used for PET imaging. By comparing the average FDG uptake activity in the area (SUV mean) with the maximum standardized FDG uptake value (SUV max), the aggressiveness of the tumor can be determined. The luminal B and triple-negative subtypes had higher SUVmax values compared to luminal A. This method showed superiority to MRI in predicting molecular subtypes, but MRI seemed to be better than PET/CT in evaluating the pathological tumor volume [80].

## 4. Conclusions

The aim of this paper was to compile and review a collection of original specialty articles, emphasizing and revising the latest correlations between the well-known molecular subtypes of BC and their mammographic, ultrasonographic, and MRI features.

By continuously seeking to expand our understanding of the early imaging signs of these tumor types and enhancing the standards of imaging evaluation methods, we can advance our knowledge in BC pathology and improve the management of this disease.

## Figures and Tables

**Table 1:** Sensitivity and specificity of different imaging screening methods when compared to the gold standard (histopathological exam). Abbreviations: MG = mammography; US = ultrasonography; ES = elastography.

HISTOPATHOLOGICAL EXAM- GOLD STANDARD		
SENSITIVITY	MG + US	99.26%
	MG	97.73%
	US + ES	88.46%
SPECIFICITY	MG+ US	44.37%
	MG	81.73%
	US + ES	67.8%

**Table 2:** Observed MRI characteristics of the two main histological types of breast cancer.

IDC (Invasive Ductal Carcinoma)	ILC (Invasive Lobular Carcinoma)
**Irregular shape**	Absence of smooth margins
**Non-circumscribed appearance**	Absence of rim-shaped enhancement
**Heterogeneous/rim enhancement**	Single spiculated non-homogenous
**Intratumoral high signal**	Mass/dominant lesion surrounded by many small enhancing foci—tendency to multifocality
**Peritumoral edema on T2 WI**	
**High peak enhancement**	

**Table 3:** Molecular subtypes of breast cancer and their prognosis. Abbreviations: ER = estrogen receptor; PR = progesterone receptor; HER2 = human epidermal growth factor 2; Ki-67 = marker of proliferation Kiel 67.

MOLECULAR SUBTYPE	PROGNOSTIC
**Luminal A** (ER+ and/or PR+, HER−)	-Best prognosis of all subtypes -Low Ki67 expression -Good response to hormonal treatment
**Luminal B** (ER+ and/or PR+, HER+)	-Less favorable prognosis compared to Luminal A -High Ki67 expression -Good response to chemotherapy
**HER2 enriched** (ER−, PR−, HER2+)	-Poor perspective -Likely high-grade -Aggressive course -Anti-HER2 therapy improves prognosis
**Triple Negative** (ER−, PR−, HER2−)	-Worst prognosis -More aggressive -High recurrency -More common in younger women -Complex management necessary

## Data Availability

Further information concerning the present article is available from the corresponding author upon reasonable request.
